# Exploring the role of histone deacetylase and histone deacetylase inhibitors in the context of multiple myeloma: mechanisms, therapeutic implications, and future perspectives

**DOI:** 10.1186/s40164-024-00507-5

**Published:** 2024-04-23

**Authors:** Jingjing Pu, Ting Liu, Xuzhen Wang, Amit Sharma, Ingo G. H. Schmidt-Wolf, Liping Jiang, Jian Hou

**Affiliations:** 1 Department of Integrated Oncology, Center for Integrated Oncology (CIO) Bonn, University Hospital Bonn, 53127 Bonn, NRW Germany; 2 Translational Biogerontology Lab, German Center for Neurodegenerative Diseases (DZNE), 53127 Bonn, NRW Germany; 3 Renji Hospital, Shanghai Jiao Tong University School of Medicine, Shanghai, 200127 China; 4 Wuxi Maternity and Child Health Care Hospital, Affiliated Women’s Hospital of Jiangnan University, Wuxi, 214002 Jiangsu China

**Keywords:** Histone deacetylase, Multiple myeloma, Histone deacetylase inhibitors, Tumor progression, Immunotherapy

## Abstract

Histone deacetylase inhibitors (HDACis) are a significant category of pharmaceuticals that have developed in the past two decades to treat multiple myeloma. Four drugs in this category have received approval from the U.S. Food and Drug Administration (FDA) for use: Panobinonstat (though canceled by the FDA in 2022), Vorinostat, Belinostat and Romidepsin. The efficacy of this group of drugs is attributed to the disruption of many processes involved in tumor growth through the inhibition of histone deacetylase, and this mode of action leads to significant anti-multiple myeloma (MM) activity. In MM, inhibition of histone deacetylase has many downstream consequences, including suppression of NF-κB signaling and HSP90, upregulation of cell cycle regulators (p21, p53), and downregulation of antiapoptotic proteins including Bcl-2. Furthermore, HDACis have a variety of direct and indirect oxidative effects on cellular DNA. HDAC inhibitors enhance normal immune function, thereby decreasing the proliferation of malignant plasma cells and promoting autophagy. The various biological effects of inhibiting histone deacetylase have a combined or additional impact when used alongside other chemotherapeutic and targeted drugs for multiple myeloma. This helps to decrease resistance to treatment. Combination treatment regimens that include HDACis have become an essential part of the therapy for multiple myeloma. These regimens incorporate drugs from other important classes of anti-myeloma agents, such as immunomodulatory drugs (IMiDs), conventional chemotherapy, monoclonal antibodies, and proteasome inhibitors. This review provides a comprehensive evaluation of the clinical efficacy and safety data pertaining to the currently approved histone deacetylase inhibitors, as well as an explanation of the crucial function of histone deacetylase in multiple myeloma and the characteristics of the different histone deacetylase inhibitors. Moreover, it provides a concise overview of the most recent developments in the use of histone deacetylase inhibitors for treating multiple myeloma, as well as potential future uses in treatment.

## Introduction

Multiple myeloma (MM) is a hematologic malignancy defined by the development of aberrant clonal plasma cells in the bone marrow, which can cause severe bone lesions, renal damage, anemia, and hypercalcemia [1]. MM is most prevalent in industrialized countries, particularly in Australia, Western Europe, and the United States, where it has the greatest prevalence [2]. It is the second most common hematologic malignancy in the United States, accounting for around 1.8% of all cancers and approximately 10% of hematologic malignancies [3]. In 2022, according to the American Cancer Society, about 34,470 new MM cases will be diagnosed in the United States, with an estimated 12,640 deaths [4]. MM is a neoplasm of older adults, with the median age of diagnosis in the United States being 69, and the median age of death is 75. Globally, men are around 1.5 times more likely than women [5]. Although recent therapies have led to a significant increase in the illness's 5-year survival rate, which now exceeds 5 years, and have improved the quality of life for patients, it is important to note that the condition is still incurable [6].

The notable enhancements in results have been correlated with the extensive utilization of autologous stem cell transplantation (ASCT) as a customary practice for eligible patients [7], along with the advancement and authorization of many innovative medications and treatment plans for managing MM [8]. In the past twenty years, various new types of drugs have been developed, including proteasome inhibitors, immunomodulatory drugs, monoclonal antibodies (mAbs), antibody–drug conjugates (ADC), bispecific T-cell engagers (BiTE), chimeric antigen-T-cell therapy (CAR-T), peptide-drug conjugates, selective inhibitors of nuclear export, and small-molecule targeted therapies [9]. With the introduction of these new treatments, treatment paradigms for MM patients have evolved as well, by employing more intricate methods, such as the use of triple therapy as opposed to dual therapy, and the increased implementation of continuous or long-term treatment, patient results can be improved. Nevertheless, the effectiveness of these treatments is frequently compromised by the emergence of resistance and the occurrence of relapse, thereby emphasising a significant deficiency in the therapy continuum [10–12]. Hence, the significance of novel therapeutic approaches for multiple myeloma cannot be overstated.

Over the last two decades, histone deacetylases (HDACs) have emerged as important therapeutic targets in cancers, particularly multiple myeloma [13, 14]. HDACis have gained significant interest as they target HDAC, which have been identified as crucial in the development of new therapy approaches for this specific condition. The fact that HDACis reduce multiple myeloma cell survival and proliferation through different mechanisms has contributed to their effectiveness. As it turns out, many HDACis have been used and evaluated in both preclinical and clinical contexts. Significantly, the FDA has granted approval to four HDACis: Vorinostat, Romidepsin, Panobinostat, and Belinostat. These HDACis are mostly utilized in clinics for hematologic tumors with less severe side effects [15]. These drugs' clinical data will be summarized later in this study.

This review provides a comprehensive analysis of the crucial role of HDACis in MM, as well as the clinical evaluation of different HDACis. It focuses on the many consequences of inhibiting histone deacetylation in MM and examines the justification for using HDACis in conjunction with medications or immunotherapies that target other pathways, with the goal of enhancing their effectiveness. Furthermore, it examines the mechanisms behind resistance to histone deacetylation inhibition and explores potential strategies to overcome this resistance through combination treatment.

In the end, it offers an in-depth review of the clinical effectiveness and safety data for treatments based on HDACis in various treatment scenarios for MM, highlighting the significance of these drugs as the primary form of treatment for MM.

### Rationale for targeting HDACs in MM

**Fig. 1:**
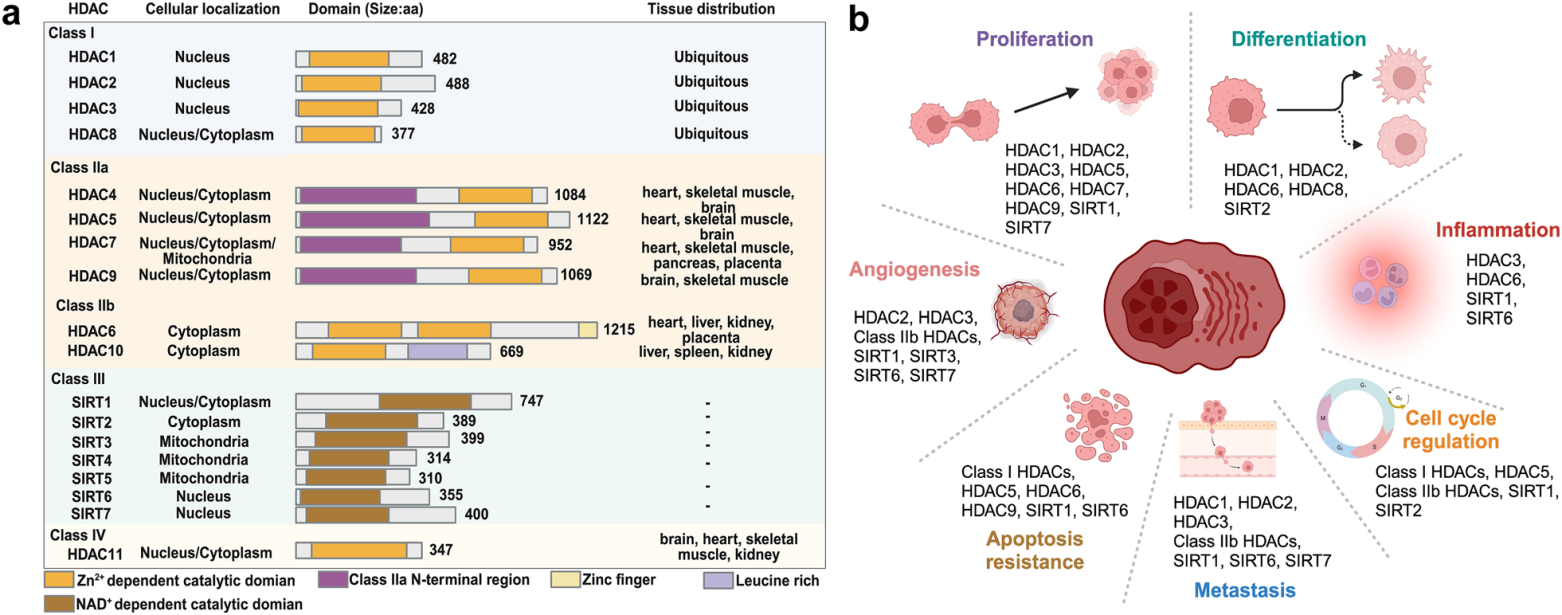
**a** Classification of HDAC family; **b** The role of HDACs in MM. Figure created with BioRender.com

Based on homology to yeast HDAC, subcellular localization, and noncellular enzymatic activity, the 18 HDAC isoforms in humans are divided into four groups, classes I (HDAC1, HDAC2, HDAC3, HDAC8), Class IIa (HDAC4, HDAC5, HDAC7, HDAC9), Class IIb (HDAC 6, DAC10), Class III (SIRT1-SIRT7), and Class IV (HDAC11) (Fig. 1a) [16–19]. Class I, II, and IV HDACs possess a deacetylase domain that relies on the presence of Zn^2+^, while class III HDACs contain a deacetylase domain that depends on the presence of NAD^+^. Class I members exhibit widespread expression, with nuclear localization being the predominant pattern. They also have an N-terminal catalytic domain and are made up of about 400 amino acids. Their catalytic domain is formed by two neighboring histidine residues, two aspartic acid residues, and a tyrosine residue centered on a Zn^2+^ ions [20, 21]. Class II members exhibit enhanced specificity in expression and possess the ability to actively transport between the nucleus and the cytoplasm. Class IIa HDACs consist of 600–1200 amino acids and possess an N-terminal regulatory domain that enables interactions with tissue-specific transcription factors and corepressors [22, 23]. In the C-terminal region of Class IIb HDACs, there is another catalytic domain and a ubiquitin-binding zinc finger domain, respectively [24, 25]. The sirtuin deacetylase family (SIRT1-7) belongs to class III, however they are not functionally linked to HDAC; their deacetylase activity is based on NAD^+^ rather than Zn^2+^-dependent enzymes [26]. HDAC11, the sole member of the class IV HDAC family, is mostly found in the nucleus. The majority of its amino acid sequence is dedicated to its catalytic domain [27].

### HDAC biology

HDACs have a crucial function in controlling gene expression by altering the acetylation state of histones, which are proteins involved in the packaging and organization of DNA in the cell nucleus [28, 29]. In the context of MM, HDACs have been associated with several facets of the disease (Fig. 1b), including cell cycle regulation [30], apoptosis resistance [31], and interactions with the tumor microenvironment (Proliferation, differentiation, inflammation, metastasis, angiogenesis) [32–34]. Notably, endothelial cells play a crucial role in the process of angiogenesis, which involves the development of new blood vessels. This process is essential for the growth and dissemination of tumors. In the microenvironment of MM, these cells undergo alterations in their properties and concurrently promote angiogenesis, thereby expediting the advancement of the disease and the development of medication resistance. HDACis have become a prominent inhibitory factor in this process by compromising the activities of endothelial cells and affecting the blood supply network of the tumor [35, 36]. Their mechanism of action involves the inhibition of HDACs, which induces alterations in gene expression in endothelial cells, ultimately leading to anti-angiogenic effects [37]. The integration of endothelial cell targeting and angiogenesis in the treatment of MM is a promising approach to overcome drug resistance and improve therapeutic results.

**Fig. 2:**
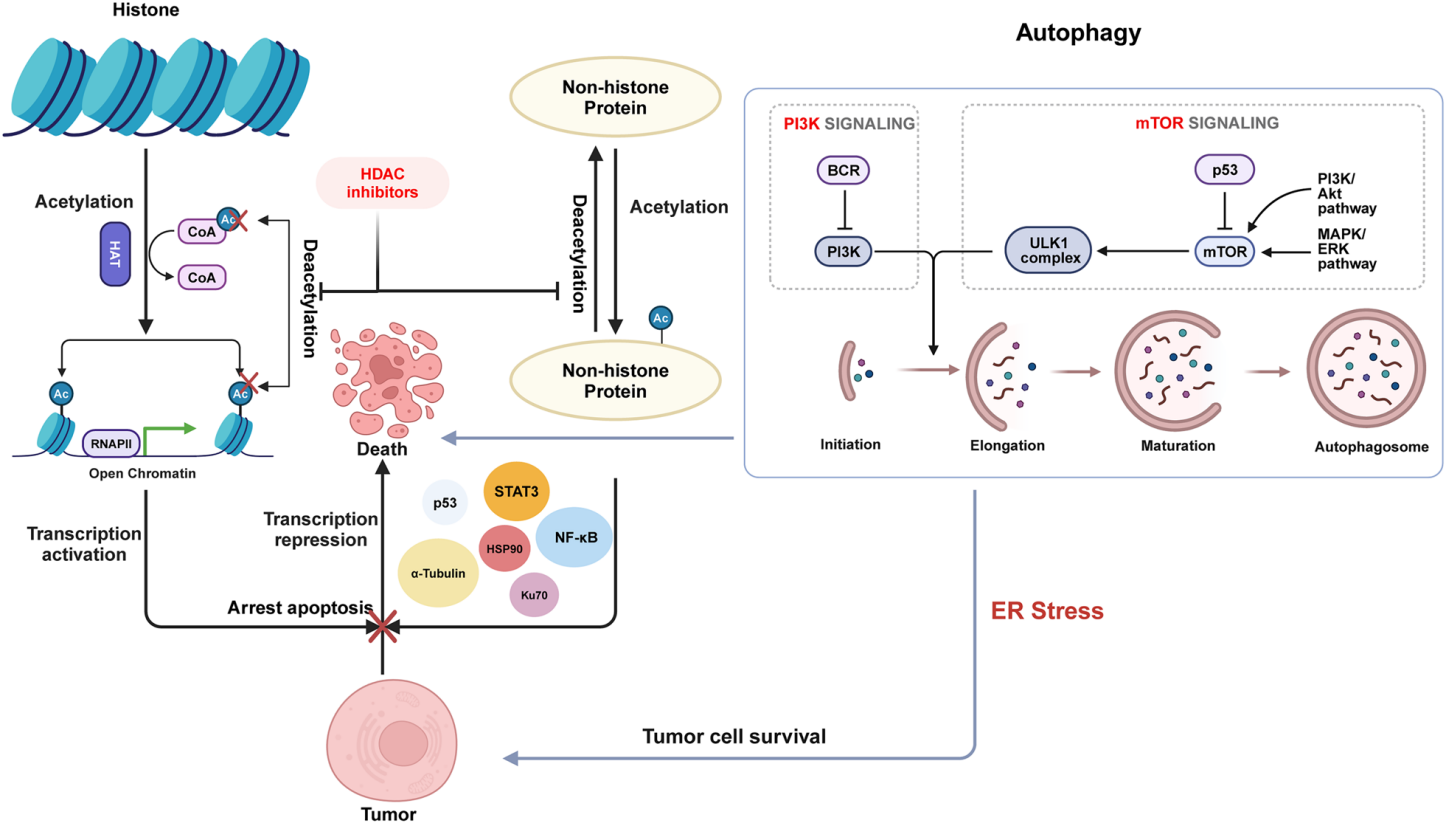
Acetylation of lysine in histone and non-histone proteins. Histone acetylation causes a loose chromatin structure, which causes gene expression. Additionally, the double-edged sword role of autophagy in tumor development and progression. Figure created with BioRender.com

HDACs, as a whole, facilitate the elimination of acetylation from lysine residues in target proteins [30, 38]. They play a critical role in regulating cell function, not only by removing acetyl groups from lysine residues on core histones, leading to tighter chromatin and reduced gene expression [14], but also by deacetylating non-histone proteins such as the tumor suppressor p53 [39–41], STAT3 [42], HSP90 [43], and NF-κB [44]. This action significantly affects these proteins' function, interactions, and stability, influencing various cellular activities [45] (Fig. 2). In MM, this regulation becomes particularly important. The constant activation of the NF-κB pathway [46] and other cancer-promoting mechanisms leads to fast cell growth and a supportive environment in the bone marrow [47]. This creates a cycle that helps MM cells survive and multiply. HDACis can break this cycle. They change the acetylation pattern of both histone and non-histone proteins, which impacts chromatin structure, gene activity, and critical signaling pathways, such as NF-κB, PI3K/AKT/mTOR, and MAPK [48, 49]. As we mentioned before, by also affecting the tumor environment and promoting cell death and cell cycle arrest, HDACis show strong potential against MM. Their ability to target both epigenetic and non-epigenetic factors highlights their promise in MM treatment, especially when used alongside other therapies [30]. Moreover, autophagy, an essential cellular mechanism responsible for the degradation and recycling of impaired organelles and proteins, assumes a multifaceted and ambivalent role in the pathology of MM [40, 50, 51]. This process facilitates cellular survival under conditions of stress by provisioning vital nutrients and energy, thereby contributing to the development of drug resistance. Conversely, aberrant or excessive autophagy may precipitate cellular demise, potentially amplifying the efficacy of anti-cancer therapeutics [52] (Fig. 2). HDACis are observed to modulate autophagy within MM cells through a bifurcated mechanism: initiating protective autophagy that favors cellular survival or provoking cytotoxic autophagy, culminating in cellular mortality [53, 54]. This comprehensive approach aims to disrupt the key cellular processes that MM cells rely on to survive and grow. In conclusion, factors such as autophagy, drug resistance, and endothelial cells are interrelated factors that influence the efficacy of MM treatment [55], especially in the context of HDAC inhibition. Understanding the complex interplay between these factors can help guide the development of new treatment strategies and improve outcomes for patients with MM.

### HDAC inhibitors

**Fig. 3:**
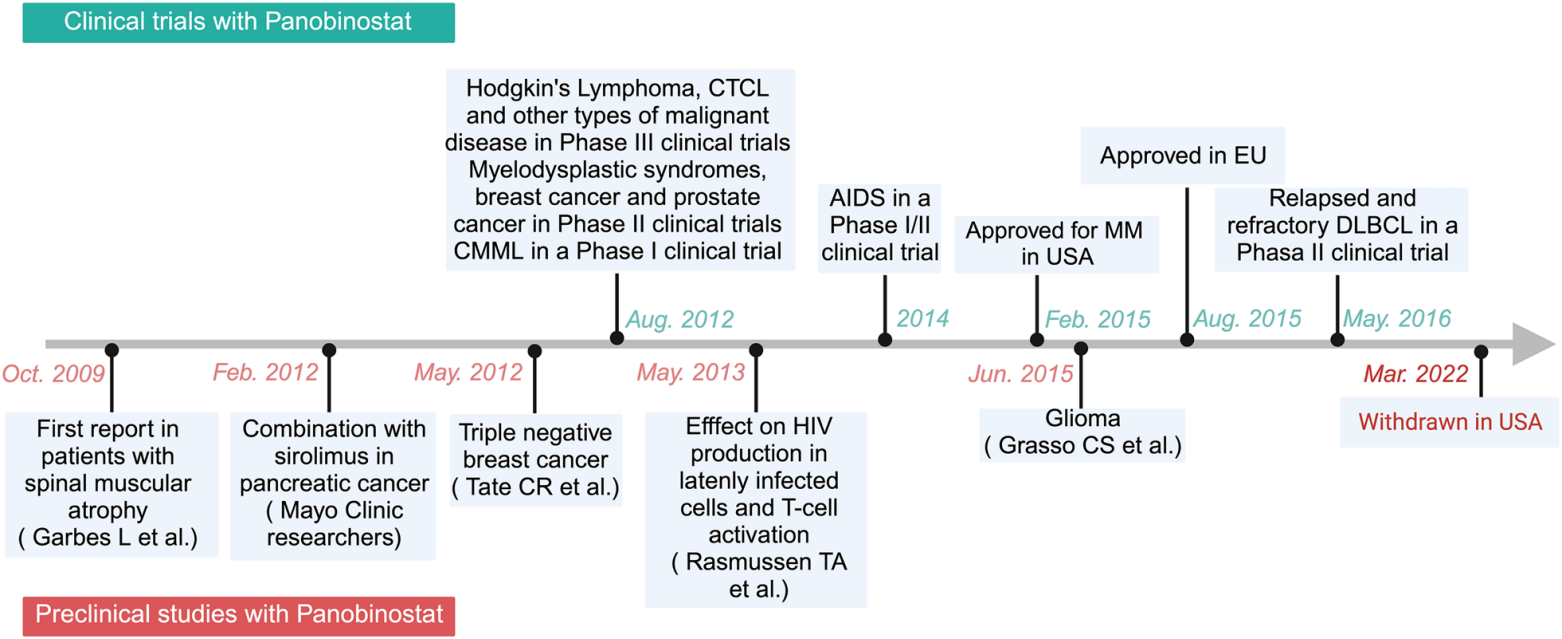
Highlights in the development of panobinostat which was firstly approved by the FDA to treat RRMM. Figure created with BioRender.com

A range of HDACis have been investigated in the context of malignancies. HDACis are categorized into six types based on their chemical structure. Short-chain fatty acid, hydroxamic acid, benzamide, cyclic peptide, mercaptoketone, sirtuin inhibitors, and other compounds [56, 57] (Table 1). Non-selective HDACis have the ability to inhibit various HDAC isoforms. However, previous research has shown that the primary focus of clinically important HDACis are HDAC 1, 2, 3, and 6. These findings indicate that the primary mechanism behind the anti-tumor properties of non-selective HDACis is the inhibition of class I and class IIb HDAC enzymes [58]. Clinical development of HDACis continues benefiting a growing number of patients with RRMM. Among them, Panobinostat (LBH589) is a strong non-selective oral pan-histone deacetylase inhibitor with efficacy in myeloma patients [59]. Panobinostat was approved by the FDA in 2015 to treat RRMM based on promising preclinical and clinical research. However, it was withdrawn in the United States in March 2022 (Fig. 3). As clinical studies progress, an increasing number of HDACis are becoming viable options for treating RRMM. For instance, Qusinostat, Gavinostat and Rocilinostat employed exclusively in the management of solid tumors and refractory leukemia, demonstrate potential efficacy in the treatment of RRMM [60, 61].

**Table 1:** Characteristics of HDACis in MM (selected)

Chemical class	Drug name	Approved by the FDA	In Phase I/II/III clinical trials	Reported targets (HDAC)	Type of cancer targeted against
Short-chain fatty acid	Valproic acid (VPA)		III	Class I, IIa	Cervical and ovarian
	Sodium butyrate		II	Class I, IIa	Colonic cancer
	Penyl butyrate		II	Class I, IIa	Urea cycle disorders
Hydroxamic acid	LBH589 (Panobinostat)	Approved for MM in 2015 (withdrawn in 2022)	III	Class I, II, IV	MM and CTCL
	Trichostain-A (TSA)		Preclinical	Class I, II, IV	Cervical and hepatoma
	SAHA (Vorinostat)	Yes (USA) Approved for CTCL	I/II	Class I, II, IV	CTCL
	JNJ-26481585 (Qusinostat)		I/II	Class I, II, IV	RRMM and solid tumors
	ITF2357 (Gavinostat)		II	Class I, II	Refractory leukemia and RRMM
	PXD101 (Belinostat)	Yes (USA) Approved for PTCL		Class I, II, IV	PTCL and RRMM
	NVP-LAQ824 (Dacinostat)		I	Class I, II	NSCC and colonic cancer
	Suberoylanilide bis-hydroxamic acid (SBHA)			Class I	Melanoma and sarcoma
	RAS2410 (Resminostat)		I/II	Class I, II	Hodgkin lymphoma and HCC
	ACY-1215 (Rocilinostat)		I	HDAC6	RRMM
	CR-2408			Class I, II, IV	MM
	Practinostat		II	Class I, II, IV	Prostate cancer
	CHR-3996 (Nanatinostat)		I	Class I	Refractory metastatic solid tumors
Benzamide	MGCD-0103 (Mocetinostat)		II	Class I, IV	Hodgkin lymphoma
	SNDX-275 (MS-275, Entinostat)		II/III	Class I	Leukemia, colorectal, gastric, pancreatic, lung, ovarian, MM
	CI-994 (Tacedinaline)		III	Class I	Pancreatic cancer, NSCC, MM, leukemia
	4SC-202 (Domatinostat)		I	Class I	Advanced hematological malignancies
	Chidamide (Tucidinostat)	Yes (China)	II	Class I, IIb	Solid tumors, PTCL, MM and ATLL
Cyclic peptide	Depsipeptide (FR901228, FK228, Romidepsin)	Yes (USA) Approved for CTCL	II	Class I	CTCL and RRMM
	Apicidin			Class I	Melanoma and leukemia
Mercaptoketone	KD5170			Class I, II	MM
Sirtuins inhibitors	Nicotinamide		III	Class III	Laryngeal cancer
	Sirtinol		Preclinical	SIRT1, II	
	Cambinol		Preclinical	SIRT1, II	
	EX-527		Preclinical/I/II	SIRT1, II	Huntington disease, glaucoma
Others	Tubacin			HDAC6	MM
	ACY-241 (Citarinostat)		Ib	HDAC6	MM

MM: multiple myeloma, RRMM: relapsed/refractory multiple myeloma, CTCL: cutaneous T-cell lymphoma, PTCL: peripheral T-cell lymphoma, NSCC: non-small cell lung carcinoma, HCC: hepatocellular carcinoma, ATLL: adult T-cell leukemia-lymphoma

### Mechanisms of action of HDACis

HDACis work in several ways to prevent myeloma cell survival and growth. Cancer cells, particularly MM cells, exhibit cell cycle disruption, resulting in accelerated cell proliferation. Non-selective HDACis or class I HDACis cause G0/G1 cell cycle arrest by upregulating cell cycle regulators, such as p21 (WAF1) [62, 63] and p53 [64, 65], or downregulation of antiapoptotic proteins such as Bcl-2 [66]. HDACis facilitate the restoration of regular immunological function, leading to a reduction in the excessive growth of malignant plasma cells. Furthermore, HDACis exert various direct and indirect effects on cellular DNA, resulting in oxidative damage [67]. They induce mitotic delays by bypassing the spindle assembly checkpoint. In our recent exploration, we uncovered the reciprocal relationships between the epigenetic machinery and the non-coding genome in the control of gene expression. This involved delving into the fascinating connections between HDAC6-induced lncRNA and its prospective sponge miRNA in the context of MM [68]. Simultaneously, as discussed in Section "HDAC biology", Heat-shock protein 90 (HSP90), a cellular chaperone essential for proteins involved in intracellular signaling (Her2/neu, Raf, ERK, NF-κB), is likewise inhibited by HDACis [69, 70]. For instance, the protein Hsp90, which acts as a molecular chaperone, is affected by the process of deacetylation carried out by HDAC6. Various pieces of evidence indicate that inhibiting both HDAC6 and Hsp90 at the same time leads to enhanced anti-tumor effects on various cancer cell lines. This emphasizes the advantages of creating a single compound that can target multiple molecules simultaneously [71]. As such, dual-targeting strategies against histone deacetylase are designed to enhance therapeutic efficacy while minimizing the side effects associated with broad-spectrum HDAC inhibition.

### Synergy with and resistance to HDAC Inhibition

Suppressing histone deacetylase has several effects that result in increased efficacy when combined with other chemotherapeutic and targeted therapies in MM, either via synergy or addition. Previous studies have shown that either panobinostat or vorinostat anticancer effects were increased in preclinical trials in patients with RRMM when combined with proteasome inhibitors such as bortezomib [72–76]. Both of them exhibit a synergistic impact in restraining cell proliferation and enhancing programmed cell death in MM cells [77]. The investigation further revealed that the co-administration of tubacin, a selective inhibitor of HDAC6, with bortezomib elicited a comparable outcome, concomitant with a notable augmentation in polyubiquitinated proteins [78]. In addition, the synergistic effect of panobinostat and romidepsin combined with proteasome inhibitors was also found in the MM cell mouse xenograft models in vivo [79, 80].

**Fig. 4:**
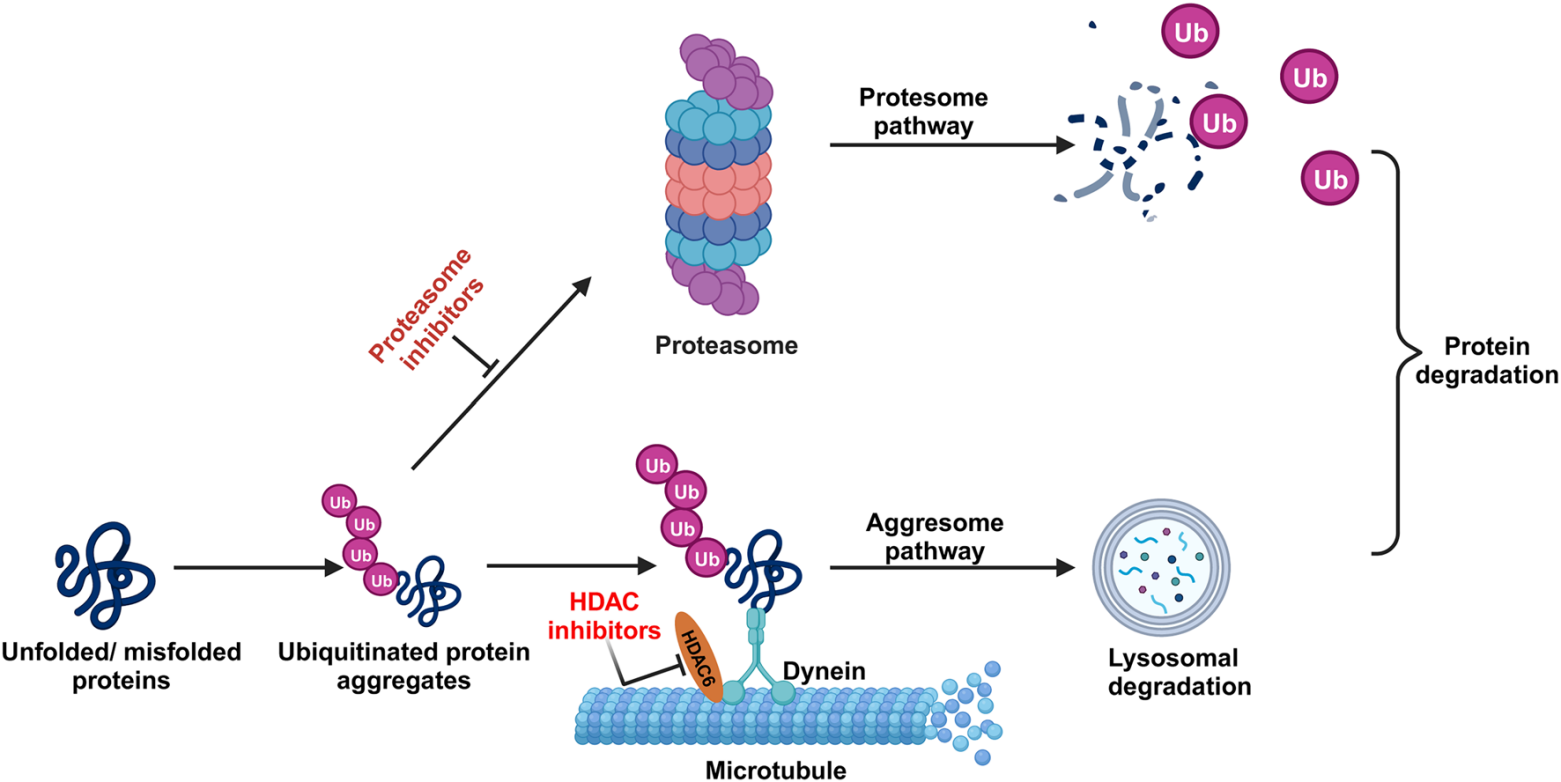
Aggresome pathway and synergy with proteasome inhibitors. Ubiquitin targets unfolded and/or misfolded proteins for destruction via the proteasome and aggresome pathways. Inhibiting proteasome pathways with inhibitors like bortezomib or carfilzomib results in the formation of ubiquitin protein aggregates, which are subsequently shuttled to the lysosome and destroyed via the aggresome pathway. Protein aggregates migrate across microtubules utilizing the dynein motor protein in the aggregation process. HDAC-6 promotes protein aggregate/microtubule complexes. If histone deacetylase (HDAC) is inhibited (together with proteasome inhibitors) at this moment, ubiquitin protein aggregates would develop further, resulting to apoptosis. If histone deacetylase (HDAC) is inhibited (together with proteasome inhibitors) at this moment, ubiquitin protein aggregates would develop further, resulting to apoptosis. Figure created with BioRender.com

The strategy of combining therapies to overcome resistance to HDACis has been demonstrated to occur through multiple mechanisms [72]. The concurrent suppression of the proteasome and aggresome pathways is the most extensively studied manifestation of synergy between proteasome inhibitors and HDACis [81, 82] (Fig. 4). The convergence of bortezomib, a proteasome-targeting agent, with an HDAC6 inhibitor, specifically directed at aggregates within tumor cells, engenders heightened accumulation of polyubiquitinated proteins, consequently inducing increased cellular stress and death [81, 82]. In particular, proteasome inhibition promotes aggregation formation, which is dependent on HDAC6 interactions with tubulin and dynein complexes. Furthermore, both proteasome inhibitors (bortezomib) and HDAC6 inhibitors (tubacin or panobinostat) enhance tubulin hyperacetylation and polyubiquitinated protein synthesis, which increases cellular stress responses and leads to autophagy and apoptosis. This is partly determined by caspase activity [81, 82]. The potential overcoming of resistance mechanisms in multiple myeloma may be achieved through the synergistic combination of HDAC inhibitors with other active agents possessing diverse mechanisms of action within the context of MM, or by incorporating novel targeted agents specifically designed to address resistance pathways, allowing the persistent use of histone deacetylase inhibition as the mainstay of the entire course of treatment.

### Clinical outcomes of HDACis in MM

Numerous studies have established the applicability of histone deacetylation inhibitors in the treatment of MM during the course of more than a decade of continuous development of HDACis. Since the FDA approved some nonselective HDACis for the treatment of MM, a growing number of HDACis have become the cornerstone of overall MM treatment and are now or are being studied as an option for induction, consolidation, and maintenance therapy, as well as a single agent or in multiple highly effective combination regimens in RRMM. Here, we summarized clinical trials involving HDACis used alone, combined with dexamethasone, immunomodulatory drugs (IMiDs), traditional chemotherapy, and novel targeted agents. It is worth noting that recent advancements in the development of HDAC inhibitors for cancer treatment are geared towards specificity and improved outcomes. Innovations include the development of class I HDAC inhibitors [83], targeting enzymes frequently overexpressed in tumors to reduce growth and offer better therapeutic options. CN133, a promising HDAC inhibitor, showcases high selectivity for class I HDACs and improved penetration into prostate tissue, hinting at enhanced efficacy in prostate cancer treatment, particularly in combination therapies [84]. Additionally, research into HDAC10 targeting has led to the creation of specific inhibitors, like Tubastatin A and its analogues, aiming for precise action against HDAC10, which is linked to poor prognosis in neuroblastoma [85]. These efforts represent a move towards more targeted cancer therapies with the potential for fewer side effects in treating MM.

### Monotherapy in MM

Wolf et al. [86] conducted a Phase II research (NCT00445068) with 38 patients with RRMM. The study used a dose of Panobinostat at 20 mg, administered three times a week, on a weekly basis within 21-day cycles. Prior to this, patients had undergone a minimum of two therapy regimens, which involved the use of an IMiD (thalidomide or lenalidomide) and bortezomib. The overall activity was deemed to be low, as seen by one partial reaction and one minimum response. Both of these responses exhibited excellent durability, lasting for 19 and 28 months, respectively. However, the trial was ended owing to insufficient efficacy. More than 80% of patients had gastrointestinal adverse events (AEs), with the bulk of these occurrences classified as grade 1–2. The most common grade 3–4 occurrences were related to blood disorders, including neutropenia, thrombocytopenia, and anaemia. Additionally, 26% of the patients reported experiencing fatigue. A Phase Ia/II dose-escalation study of oral Panobinostat was conducted on 176 patients with hematologic malignancies, including 12 with RRMM, as part of another clinical trial (NCT00621244) [87]. The doses of Panobinostat ranged from 20 to 80 mg in two different dose-escalation regimens, either administered three times per week or once every two weeks. In Phase II, the prescribed dosage for MM was 40 mg administered on a weekly basis. The maximum acceptable dose, on the other hand, was Panobinostat 60 mg given every two weeks. Coincidentally, one RRMM patient responded somewhat like adverse events, particularly gastrointestinal and hematologic AEs, were similar with those found in earlier studies. This trial confirmed overall safety and guided dosage for further monotherapy and combo treatment. In addition to Panobinostat, Vorinostat (NCT00045006), ITF2357 (NCT00792506), Entinostat (NCT00015925), Tacedinaline (NCT00005624), Domatinostat (NCT01344707) and Romidepsin (NCT00066638) were also used in monotherapy clinical trials. In summary, while Panobinostat has shown some efficacy as a monotherapy in treating MM, its clinical benefits are more pronounced and better supported when used in combination with other therapies. The management of multiple myeloma remains complex, requiring a multidisciplinary approach to optimize patient outcomes (Table 2).

**Table 2:** Clinical studies evaluating HDACis for the treatment of MM

NCT number	Phase	Patients	Regimens	HDACis dose/Schedule	Response rates	Grade 3/4 toxicities	References
NCT00445068	II	n = 38	Panobinostat	20 mg, Days 1, 3, 5, 8, 10, 12, every 21 days	ORR: 2.63%	Thrombocytopeni (50%), anemia (11.2%), neutropenia (8%), fatigue (26%)	[86]
NCT00621244	Ia/II	n = 176	Panobinostat	From 20-80 mg three times a week or once every 14 days	Not found	Thrombocytopenia (41.5%), neutropenia (21%), fatigue (21%)	[87]
NCT01720875	II	n = 16	Vorinostat/ Bortezomib/ Dexamethasone	400 mg, Days 1–14, every 21 days	ORR: 81.3%	Thrombocytopenia (50%), diarrhea (6.3%), fatigue (6.3%), anemia (6.3%)	[88]
NCT01583283	I	n = 38	ACY-1215/ Lenalidomide/ Dexamethasone	From 40–240 mg once daily to 160 mg twice daily	ORR: 55%	Fatigue (18%), diarrhea (5%)	[89]
NCT01502085	I/II	n = 25	Vorinostat/ Lenalinomide/ Dexamethasone	Vorinostat: 400 mg once a week; Lenalidomide: 25 mg once two weeks; Dexamethasone: 40 mg, Days 1, 8, 15 and 22	ORR: 24%	Thrombocytopenia (56%), fatigue (72%), diarrhea (72%), neutropenia (68%), vomiting (12%)	[90]
NCT00642954	I	n = 31	Vorinostat/ Lenalidomide/ Dexamethasone	Vorinostat: 400 m, days 1–7 and 15–21; Lenalidomide: 25 mg, days 1–21; Dexamethasone: 40 mg, Days 1, 8, 15, 22, every 28 days	ORR: 47%	Anemia (58%), thrombocytopenia (58%), diarrhea (55%), fatigue (55%), cough (45%)	[91]
NCT02290431	II	n = 31	Panobinostat/ Bortezomib/ Dexamethasone	20 mg, Days 1, 3, 5, 8, 10, 12, every 21 days	ORR: 80.6%	Thrombocytopenia (48.4%), fatigue (25.8%), diarrhea (22.6%), neutropenia (22.6%), lymphopenia (22.6%)	[93]
NCT01440582	I	n = 55	Panobinostat/ Bortezomib/ Lenalidomide/ Dexamethasone	Bortezomib: (1.3 mg/m^2^), Days 1, 4, 8, 11; Lenalidomide: 25 mg, Days 1–14; Dexamethasone: 20 mg, Days 1, 2, 4, 5, 8, 9, 11, 12	Not found	Thrombocytopenia (17%), diarrhea (17%)	[94]
NCT02654990	II	n = 248	Panobinostat/ Bortezomib/ Dexamethasone	82 to panobinostat 20 mg thrice weekly, 83 to panobinostat 20 mg twice weekly, and 83 to 10 mg panobinostat three times weekly	ORR: 62·2% (20 mg three times weekly group); 65·1% (20 mg twice weekly group), 50·6% (10 mg three times weekly group)	Thrombocytopenia (32%), neutropenia (15.2%), pneumonia (11.6%)	[95]
NCT01083602	II	n = 55	Panobinostat/ Bortezomib/ Dexamethasone	20 mg, Days 1, 3, 5, 8, 10, 12, every 21 days	ORR: 34.5%	Thrombocytopenia (63.6%), diarrhea (20%), fatigue (20%), anemia (14.5%), neutropenia (14.5%), pneumonia (14.5%)	[96]
NCT00773838	II	n = 143	Vorinostat/ Bortezomib/ Dexamethasone	400 mg, Days 1–14, every 21 days	ORR: 11.3%	Diarrhea (4.2%), asthenia (2.8%), thrombocytopenia ( 2.8%), pneumonia ( 2.1%), neuralgia (1.4%)	[97]
NCT00858234	I	n = 9	Vorinostat/ Bortezomib	400 mg, Days 1–14, every 21 days	ORR: 44%	Thrombocytopenia (100%), lymphopenia (43%), neutropenia (29%), anemia (29%), nausea (29%), dehydration (29%), pneumonia (29%), diarrhea (14%), decreased appetite (14%), fatigue (14%), hypokalemia (14%)	[101]
NCT01023308	III	n = 768	Panobinostat/ Bortezomib/ Dexamethasone/ Placebo	Panobinostat: 20 mg (hard gelatin capsules); Bortezomib: 1.3 mg/m2 as a 3 to 5 s bolus intravenous injection; Dexamethasone: 20 mg every day	ORR: 60.7%	Pneumonia (14.7%), thrombocytopenia (7.35%), diarrhea (11.29%) anemia (3.67%), vomiting (3.15%), asthenia (3.94%), fatigue (2.89%) pyrexia (4.20%),	[102]
NCT01549431	I	n = 32	Panobinostat/ Carfilzomib	20 mg, Days 1, 3, 5, 15, 17, 19, every 28 days	ORR: 63%	Thrombocytopenia (41%), fatigue (17%), nausea (12%), vomiting (12%)	[103]
NCT01496118	I/II	n = 80	Panobinostat/ Carfilzomib	20 mg, 30 mg, Days 1, 3, 5, 15, 17, 19, every 28 days	ORR: 84.4%	Thrombocytopenia (60.6%), fatigue (18.2%), anemia (12.1%), dyspnea (12.1%), diarrhea (9.1%), neutropenia (9.1%), nausea (6.1%), vomiting (6.1%), peripheral neuropathy (3%)	[104, 105]
NCT01464112	I	n = 18	JNJ-2641585 / VELCADE / Dexamethasone	JNJ-2641585: 10 mg three times weekly oral dose with VELCADE + Dexamethasone	ORR: 88.2%	Thrombocytopenia (61.1%), asthenia (55.6%), diarrhea (66.7%)	[110]
NCT00773747	III	n = 637	Vorinostat/ Bortezomib	400 mg, Days 1–14, every 21 days	ORR: 11.3%	Thrombocytopenia (45%), neutropenia (28%), anaemia (17%)	[111]
NCT00532389	Ib	n = 62	Panobinostat/ Bortezomib	Panobinostat 20 mg thrice weekly every week + Bortezomib 1.3 mg/m^2^, every 21 days	ORR: 73.3%	Thrombocytopenia (85.1%), neutropenia (63.8%), asthenia (29.8%), fatigue (20.0%)	[112]
NCT00742027	II	n = 27	Panobinostat/ Lenalidomide/ Dexamethasone	20 mg, Days 1, 3, 5, 15, 17, 19, every 28 days	ORR: 41%	Neutropenia (59%), thrombocytopenia (31%), fatigue (12.5%), infection (15.6%), diarrhea (9.4%) anemia (5%)	[113]

ORR: overall response rate, data sourced from the clinicaltrials.gov site

### Doublet combination therapy with dexamethasone

The preclinical research demonstrated the synergistic effects of HDACis in combination with bortezomib and dexamethasone in MM cell lines. Additionally, the safety data from monotherapy provided a foundation for conducting combination studies (Table 2). These trials ultimately resulted in the accelerated approval of the treatment regimen [82]. In a phase II study (NCT01720875) [88], 16 MM patients, previously treated once, received a regimen of bortezomib, dexamethasone, and vorinostat, showing an 81.3% overall response rate with 100% clinical benefit. Despite a median progression-free survival of 11.9 months and maintenance treatment with vorinostat, 75% of the participants required dose adjustments or discontinued treatment due to side effects. The findings reveal that, although toxicity and dosage reductions were challenges, this combination therapy is effective in treating relapsed myeloma. This success underscores the importance of continuing to refine HDAC inhibitor-based combinations, aiming to improve both their tolerability and efficacy for myeloma treatment. Between July 2012 and August 2015, a study (NCT01583283) enrolled 38 patients to test ricolinostat [89]. Yee et al. found ricolinostat to be mostly safe, with the best dose determined as 160 mg once daily for 21 days in a 28 day cycle, combined with two other medications. The most common side effects were mild to moderate fatigue and diarrhea. The drug effectively inhibited its target enzyme without significantly affecting other enzymes, and its effectiveness wasn’t compromised when taken with the other medications. In early assessments, 55% of patients showed a positive response to the treatment, suggesting ricolinostat could be a promising option for patients with RRMM. The studies (NCT01502085 and NCT00642954) explored a new combination therapy of vorinostat, lenalidomide, and dexamethasone for treating MM, based on promising lab research. It was a phase I trial involving patients with RRMM, aiming to find the highest dose patients could tolerate without severe side effects. The maximum dose tested was well-tolerated, with drug-related adverse events in 90% of patients and serious ones in 45%. About 47% of participants showed a partial or better response to the treatment, indicating the combination's potential effectiveness with manageable side effects [90, 91]. In a study (NCT01023308) conducted between January 2010 and February 2012 involving 768 patients with RRMM, participants were divided into two groups: one received a combination of panobinostat, bortezomib, and dexamethasone (387 patients), and the other received a placebo with bortezomib and dexamethasone (381 patients). The panobinostat group showed a significantly longer median progression-free survival of nearly 12 months compared to 8 months in the placebo group. Although overall survival rates were not conclusive, the panobinostat group had a slightly higher median overall survival at the time of analysis. The study also found a higher rate of complete or near complete response in the panobinostat group compared to the placebo group, though overall response rates (ORR) were similar. The panobinostat group experienced more serious adverse events and grade 3–4 laboratory abnormalities. The findings suggest panobinostat could be beneficial for treating this patient population, but longer follow-up is needed to assess the impact on overall survival [92]. Furthermore, more and more clinical trials show that Doublet combination therapy with dexamethasone can improve the efficacy of treatment in RRMM [93–97].

### Combination therapy with IMiDs

Due to encouraging preclinical anti-MM action, the effectiveness of HDACis has been investigated in combination with other treatments, such as IMiDs (Table 2). Specifically, panobinostat has been used in combination with lenalidomide and dexamethasone. The Phase I clinical trial (NCT01440582) demonstrates the safety and efficacy of combining VRd (Bortezomib plus lenalidomide and dexamethasone) with a 10 mg dose of panobinostat in newly diagnosed multiple myeloma patients who are eligible for transplantation. In early testing, the lowest dose did not cause serious side effects, while a higher dose did in two patients, indicating it was too strong. Therefore, the study established the lower dose as the safest and most tolerable for patients. This combination therapy shows promise for treating newly diagnosed multiple myeloma in patients eligible for a transplant, but more extensive research is needed to confirm these findings [94]. Between July 2012 and August 2015, a study (NCT01583283) enrolled 38 patients to assess the safety and efficacy of ricolinostat in treating MM. The study identified a recommended dose of ricolinostat at 160 mg daily for future research, following two cases of significant adverse effects at a higher dosage. Common side effects included fatigue and diarrhea, but the drug demonstrated a promising ability to selectively inhibit HDAC6 without significantly impacting HDAC1, suggesting it could enhance treatments with lenalidomide and dexamethasone. Preliminary results showed a 55% response rate among participants, indicating that ricolinostat could be a safe and effective option for RRMM [89]. Moreover, the Phase I/II clinical trial (NCT01502085), and the Phase I clinical trial (NCT02569320) demonstrate that vorinostat, and AR-42 have the potential to synergize with lenalidomide and dexamethasone, hence improving their effectiveness in RRMM [89, 90, 98].

### Combination therapy with conventional chemotherapy

In the 1980s, the primary therapeutic choices for MM were induction therapy utilizing alkylating agents such anthracyclines and steroids, as well as high-dose chemotherapy followed by autologous stem cell transplantation. As previously stated, the introduction of advanced medicines, including proteasome inhibitors, immunomodulatory drugs, monoclonal antibodies, and histone deacetylase inhibitors, has led to a notable enhancement in prognosis through the use of a new therapy strategy. Multiple treatment protocols including these innovative medications in different combinations have been formulated and assessed in clinical trials. Annually, the outcomes of these novel therapeutic regimens are disseminated through publication. In the context of this multifaceted contemporary landscape, conventional chemotherapeutic agents persist in retaining prominence, particularly when integrated with emerging therapeutic modalities [99]. We reviewed clinical trials of HDACis in combination with conventional chemotherapy, among them, we found only two ( NCT00744354 and NCT01394354) and were unable to track the results.

### Combination therapy with novel targeted agents

As elucidated in Section “Synergy with and resistance to HDAC Inhibition”, proteasome inhibitors exhibit synergistic effects, concurrently impeding cellular proliferation and augmenting apoptosis in MM cells [77]. We found that Bortezomib, Carfilzomib, and Ixazomib were predominantly used in clinical trials (Table 2). Bortezomib is a specific and reversible inhibitor of proteasomes. It works by directly attaching to the β1 and β5 subunits of the catalytic 20S complex, hence preventing chymotrypsin-like activity85. Treatment with bortezomib enhances the bone marrow microenvironment by stimulating the development of osteoblasts and decreasing the activity of osteoclasts that depend on the receptor activator of NF-κB (RANKL). This effect is achieved through the activation of NF-κB, p38, and AP-1 pathways, and is influenced by the dosage of bortezomib [100]. The Phase I clinical trial study (NCT00858234) revealed that the most predominant adverse events were thrombocytopenia, leukopenia, neutropenia, diarrhea, nausea, decreased appetite, and vomiting [101]. Another Phase II clinical trial study (NCT01720875) showed that despite observed toxicity and dose reductions, which demonstrated that the combination of vorinostat, bortezomib, and dexamethasone was effective and had good response rates in relapsed myeloma, suggesting further optimization of HDAC inhibitor-based combination therapy for myeloid Tumor to improve tolerance and enhance efficacy [88]. However, the findings from the Phase III clinical trial study (NCT01023308) revealed that panobinostat was linked to a marginal improvement in overall survival when juxtaposed with the combination of bortezomib and dexamethasone placebo. Optimized regimens have the potential to prolong therapeutic duration and enhance patient outcomes; however, additional trials are requisite to corroborate these observations [102]. Carfilzomib is a second-generation drug that inhibits proteasomes and is mostly used for patients with multiple myeloma who have not responded to previous treatments or have experienced a relapse. Carfilzomib inhibits chymotrypsin-like activity by attaching to the catalytic 20S proteasome. Unlike bortezomib, this interaction is permanent and more specific, which accounts for certain side effects that are absent in bortezomib therapy. The usual route of administration for carfilzomib is intravenous, with a frequency of twice per week for a period of three weeks. The recommended dose is 27 mg/m^2^. Carfilzomib's molecular mode of action is similar to that of bortezomib, which includes inducing apoptosis and improving bone injury. Carfilzomib side effects may include hypertension, cardiotoxicity, thrombocytopenia, hypocalcemia, and gastrointestinal problems [103–105]. Ixazomib is an innovative proteasome inhibitor used orally at a dosage of 4 mg once per week. It functions by obstructing the enzyme in MM cells, impeding their capacity to proliferate and endure [106], nevertheless, only one clinical trial (NCT02057640) has been completed so far, but no definite results can be obtained. Common adverse effects of ixazomib encompass thrombocytopenia, edoema in the lower extremities, peripheral neuropathy (resulting in weakness, numbness, and pain in the hands and feet), gastrointestinal disturbances such as diarrhoea, constipation, nausea, vomiting, and back pain [107].

### The clinical safety of HDAC inhibitors in MM

**Fig. 5:**
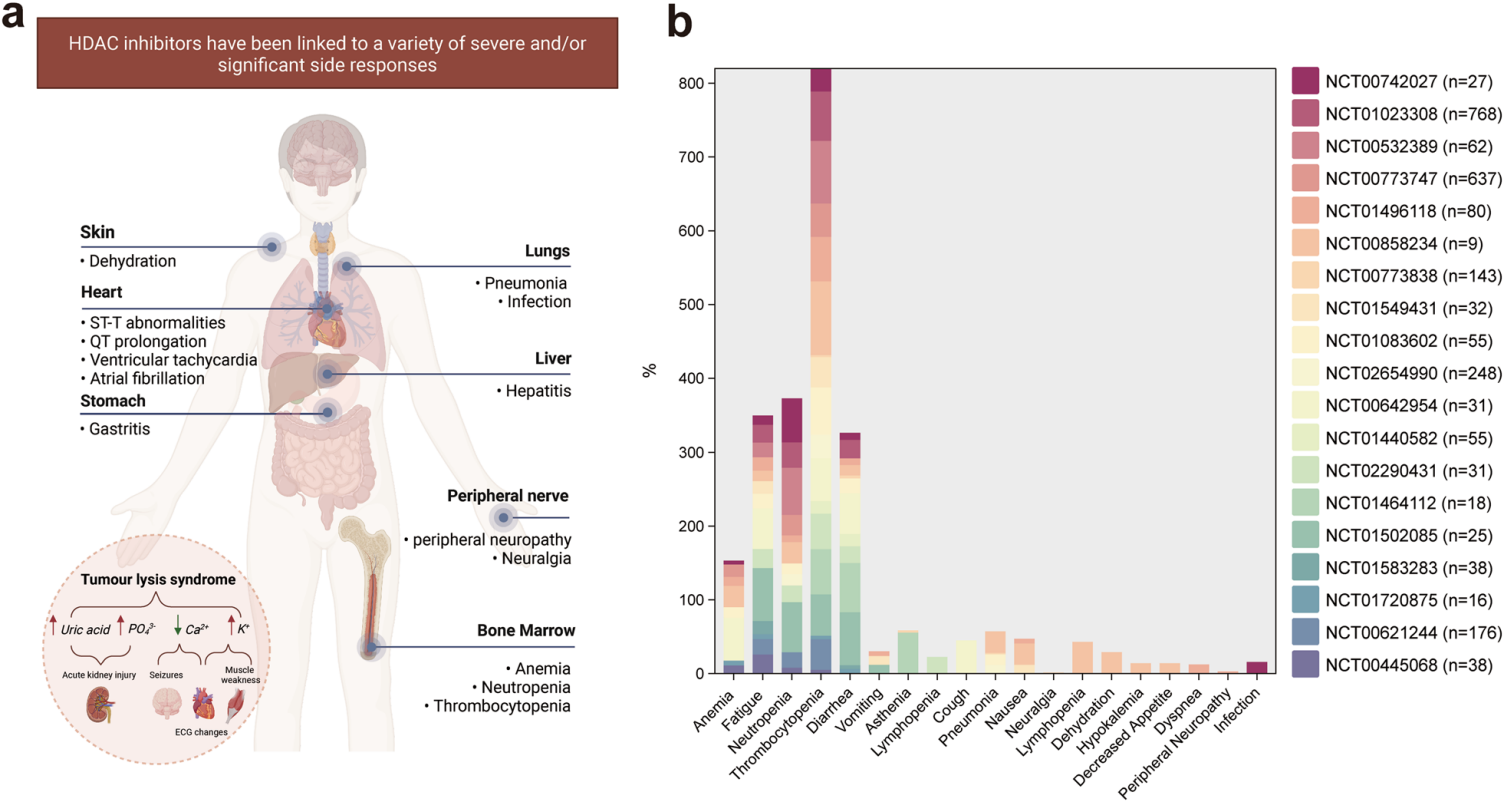
**a** HDAC inhibitors have been linked to a variety of severe and/or significant side responses; **b** Distribution of grade 3/4 toxicities in clinical trials (Table 2) of histone deacetylase inhibitors. Figure created with BioRender.com

There is an overexpression of HDAC in cancer cells, and the use of HDACis has been shown to enhance the outcomes of individuals who have been diagnosed with haematological malignancies include T-cell lymphomas and multiple myeloma. Five drugs were previously approved in different national jurisdictions, namely belinostat, chidamide, romidepsin, vorinostat and Panobinostat. It is worth noting that Secura Bio, Inc. requested the withdrawal of FDA approval for Panobinostat in 2021, citing the impracticality of conducting necessary postmarketing trials. Subsequently, in March 2022, the FDA withdrew panobinostat from the US market [108]. However, despite its removal from the US market, panobinostat continues to be employed in Europe as a viable treatment option for patients whose diseases have advanced after undergoing standard therapies. These drugs have been linked to a variety of severe and/or significant side responses, including myelosuppression, diarrhea, hepatic effects and various cardiac effects [109]. In this section, we have selected the most important side effects for review (Table 2, Fig. 5a).

### Myelosuppression

From Fig. 5b, we can see five medication clinical studies revealed 3 common side effects including thrombocytopenia, neutropenia and anemia. Thrombocytopenia is common and can result in bleeding, although neutropenia is frequently a sign of infection. These side effects may be sufficiently serious to necessitate the transfusions of blood and/or the administration of granulocyte colony-stimulating agents. To reduce the clinical effects, blood counts should be checked on a frequent basis and dose modifications done as needed; nonetheless, if toxicities of grade 3 or 4 return after reducing the dosage, treatment should be discontinued. In the aggregate, the majority of clinical trials have demonstrated myelosuppression as a noteworthy side effect, warranting careful consideration.

### Cardiac effects

The ether-a-go-go (hERG) channel in humans is responsible for controlling the duration of ventricular repolarization, which is visually represented as the QT interval on the surface electrocardiogram (ECG). Drugs that inhibit or reduce the function or expression of hERG channels lead to an elongation of the QT interval. Torsades de pointes (TdP), a potentially fatal ventricular tachyarrhythmia, can occur when the QTc interval is extended due to excessive duration or the presence of risk factors. Schiattarella et al. [114] discovered that HDACis elicit typical albeit insignificant cardiac side effects, mostly manifesting as ECG abnormalities such as ST-T abnormalities and QT prolongation. This conclusion was drawn after analysing 62 trials with a collective patient population of 3268 individuals. The most common electrocardiographic abnormalities seen in patients treated with romidepsin (25.3%) and panobinostat (22.3%) were ST depression and/or T wave inversion, which accounted for 14.5% of the patients. QTc prolongation was observed in 4.4% of the total 3268 individuals. This percentage was lower than the rates reported for belinostat (12.2%), panobinostat (4.3%), vorinostat (3.4%), and romidepsin (3.3%). Ventricular tachycardia was observed in 0.6% (21/3268) of the entire study group, with the majority of cases occurring after the administration of romidepsin (19/944, 2.0%) or panobinostat (2/1047, 0.2%). Treated persons exhibited atrial fibrillation, whereas 13 individuals (0.4%) reported experiencing atrial fibrillation. This was mostly detected in vorinostat (8/888) and belinostat (2/221) patients. [109].

### Gastrointestinal effects

From Fig. 5b, It is readily apparent that gastrointestinal side effects are also one of the main side effects. A comprehensive analysis of clinical studies has indicated that the use of antiemetic and antidiarrheal medications, together with fluid and electrolyte supplements, may be necessary to manage symptoms of nausea, vomiting, and diarrhoea following therapy with any of the five treatments (Belinostat, Panobinostat, Romidepsin, Vorinostat, Chidamide). Panobinostat has the potential to induce severe diarrhoea (grade 3 or 4) in 25% of people on therapy, which may necessitate a decrease in dosage or complete cessation of the treatment.

### Hepatic effects

Complications arising from therapeutic interventions with romidepsin, panobinostat, belinostat, and chidamide have been systematically documented, frequently manifesting as elevated blood transaminases and/or bilirubin levels. Notably, vorinostat has not been correlated with any hepatic side effects. Despite a comprehensive literature search yielding no reports of clinically significant hepatotoxicity associated with these agents, a pivotal event in a belinostat clinical study, marked by a treatment-related fatality linked to hepatic failure, prompted the FDA to modify the approved label for belinostat. The revised label underscores the potential for fatal toxicity and advocates for pre-treatment and cyclical liver function test monitoring, it is particularly important. In the event of discernible hepatic impairment, a judicious course of action involves either dose adjustment or discontinuation, contingent upon the severity of the observed hepatotoxicity [109].

### Agent‐specific adverse effects

Table 3 concisely summarizes distinct extra adverse effects linked to various HDACis that set them apart from the wider class. The infections observed with belinostat and romidepsin are most likely caused by neutropenia, while cases of hemorrhage associated with panobinostat and pericardial effusion after chidamide therapy are coupled with thrombocytopenia created by these drugs. Increased levels of creatine phosphokinase in conjunction with chidamide and the presence of cardiac ischemia with panobinostat may indicate the potential of these particular drugs to cause harm to the myocardium.

**Table 3:** The agent-specific reported adverse effects associated with HDACis

HDACi	Specific adverse effects reported in clinical trials
Panobinostat	Cardiac ischaemia, haemorrhage
Vorinostat	Hyper-glycaemia, pulmonary embolism, deep vein thrombosis
Belinostat	Infections, tumour lysis syndrome
Romidepsin	Infections, tumour lysis syndrome
Chidamide	Raised creatine phosphokinase levels, pericardial effusion

Tumor lysis syndrome, a phenomenon that often occurs in the early stages of treatment and is frequently associated with belinostat and romidepsin, is commonly seen in patients with advanced-stage disease and/or high levels of hematological tumor burden. This syndrome is a metabolic disorder that can be life-threatening. It is characterized by high levels of uric acid, potassium, and phosphate, and low levels of calcium. This condition not only causes gastrointestinal symptoms like nausea and vomiting, but also leads to serious complications such as acute uric acid nephropathy, acute kidney failure, seizures, cardiac arrhythmias, and even death.

However, the clarification of prothrombotic and hyperglycemic effects associated with vorinostat poses challenges, as these phenomena may be attributed to factors such as the investigational drug itself, the characteristics of the patient population under scrutiny, or concurrent therapeutic interventions.

HDACis are a hopeful treatment for MM, aiming to correct cancer-specific gene patterns. Yet, their effectiveness is complicated by the fact that MM patients differ greatly in their genetic makeup, leading to varied responses to these drugs. This variation highlights the need for identifying markers that can predict who will benefit most from these treatments. Additionally, the side effects of HDAC inhibitors can vary from mild to severe, making it crucial to manage these carefully to ensure patients truly benefit from the treatment. Looking ahead, research is zeroing in on finding these predictive markers, creating drug combinations that work better and have fewer side effects, understanding why some patients develop resistance, and paying closer attention to how treatments impact patients' quality of life. This approach aims to make HDACis treatment more personalized, maximizing benefits while reducing drawbacks for MM patients.

### Challenges in the combined use of HDACis and immunotherapy

**Fig. 6:**
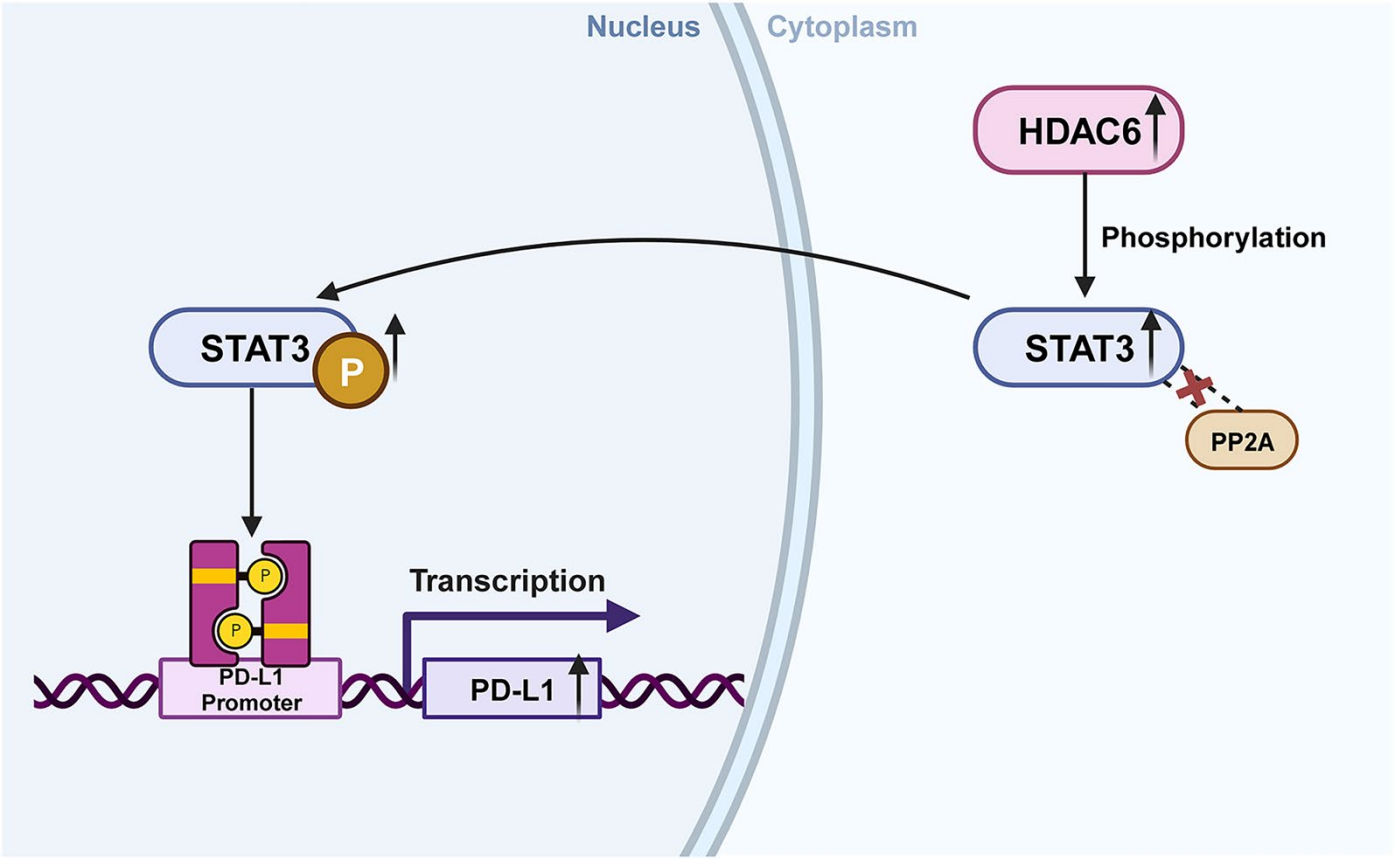
Mechanistic illustration of HDAC6 in STAT3-PD-L1 pathway: When HDAC6 levels are high, STAT3 accumulates in a phosphorylated form, reducing the interaction between STAT3 and PP2A. After entering the nucleus, pSTAT3 and HDAC6 bind to the PD-L1 promoter, promoting PD-L1 expression. Figure created with BioRender.com

Immunological evasion in cancer is a critical process that involves the expression of immunological checkpoints, including PD-1, PD-L1, and CTLA-4. Inhibiting these checkpoints is an effective approach for treating cancer. Multiple studies demonstrate that STAT3 is involved in directly or indirectly controlling these immunological checkpoint molecules [115–118]. Notably, HDAC6 emerges as a significant regulator of the STAT3 pathway [119–121]. Lienlaf et al. provided evidence that HDAC6 plays a role in the body's defence against tumours in melanoma by affecting the STAT3-PD-L1 pathway [121], this discovery was further supported by Keremu et al. in their study on osteosarcomas [120]. Elevated production of HDAC6 leads to the phosphorylation of STAT3 and its translocation into the nucleus, without causing any changes in acetylation of its co-protein PP2A. Phosphorylated STAT3 and HDAC6 coexist in the nucleus and target the PD-L1 promoter, resulting in the activation of transcription and the enhancement of PD-L1 gene expression [19, 121, 122] (Fig. 6). Notably, preclinical studies indicate that a combination of HDAC6 inhibitor and PD-L1 antibody enhances γδ T cell antitumor functions [123]. This underscores the potential of targeting the HDAC6 inhibition-PD-1/PD-L1 pathway as a novel approach to augment cancer immunotherapy. The concurrent use of pan-HDACis and cytokine-induced killer (CIK) cell treatment [124], which has demonstrated efficacy in preclinical multiple myeloma models [125, 126], provides additional validation for this idea. The presence of specific HDAC6 inhibitors such as ACY-1215, tubastatin A, and ricolinostat presents a potential opportunity for their use, either alone or in conjunction with CIK cell therapy, in medical environments. This offers a hopeful pathway for the treatment of cancer.

The combined use of HDACis and immunotherapy holds promise for enhancing cancer treatment outcomes, but it also presents several challenges. (1) Limited understanding of mechanisms: The mechanisms through which HDACis interact with the immune system and modulate responses to immunotherapy are not fully understood. Better insights into these mechanisms are crucial for optimizing combination therapies. (2) Dose-dependent effects: The effects of HDACis can be dose-dependent, and finding the right balance is critical. High doses of HDACis may have immunosuppressive effects, counteracting the desired immune activation promoted by immunotherapy. (3) Off-target effects: HDACis can affect various cellular processes beyond histone acetylation, potentially leading to off-target effects [127]. Understanding and minimizing these off-target effects is important to avoid unintended consequences on immune cells and overall treatment efficacy. (4) Patient heterogeneity: Patient responses to HDACis and immunotherapy can vary significantly. Identifying biomarkers to predict which patients are more likely to benefit from the combination is a challenge. Personalized medicine approaches may be essential for optimizing treatment strategies. (5) Toxicity and side effects: HDACis can be associated with toxicities and side effects, including hematological toxicity and fatigue. Combining these agents with immunotherapy may exacerbate these issues, and managing the overall toxicity profile is crucial for patient safety and adherence. (6) Resistance development: Tumor cells can develop resistance to HDACis and immunotherapy. Understanding the mechanisms of resistance and developing strategies to overcome or prevent resistance is essential for long-term treatment success. (7) Optimal sequence and timing: Determining the optimal sequence and timing of HDACis and immunotherapy is challenging. The order in which these treatments are administered can impact their effectiveness, and finding the right schedule is critical for maximizing therapeutic benefits. (8) Synergistic vs. antagonistic effects: Achieving synergistic effects between HDACis and immunotherapy is the goal, but there is a risk of antagonistic interactions. Careful preclinical and clinical studies are needed to assess the compatibility of these treatments and avoid potential counteractive effects. (9) Clinical trial design: Designing clinical trials to effectively evaluate the safety and efficacy of combined HDACis and immunotherapy is challenging. Robust study designs, appropriate patient selection, and relevant endpoints are necessary to draw meaningful conclusions. (10) Regulatory hurdles: Regulatory approval for combination therapies can be complex. Coordinating the approval process for two or more agents may require additional evidence of safety and efficacy, and navigating regulatory pathways is a significant challenge. Addressing these challenges will require collaborative efforts from researchers, clinicians, and regulatory authorities to advance the understanding and implementation of combined HDACis and immunotherapy for cancer treatment. Ongoing research and clinical trials are essential to further elucidate the complexities and refine treatment strategies.

## Conclusions and future directions

In the past decade, the landscape of MM treatment has undergone significant transformation, largely due to advancements in HDACis, immunomodulatory drugs, and other novel therapies. The incorporation of HDACis into the therapeutic arsenal has expanded the spectrum of effective treatment options, leading to increased patient longevity and improved quality of life. Presently, a wide array of potent therapy regimens that leverage HDACis as a backbone is available, indicating a pivotal shift in MM management strategies. Moreover, ongoing research is exploring innovative approaches, such as the integration of HDACis with monoclonal antibodies, targeted medicines, and cellular immunotherapy, aiming to further enhance treatment efficacy and patient outcomes.

A notable area of progress involves the synergistic combination of HDACis with anti-CD38 monoclonal antibodies, such as daratumumab, which received FDA approval in November 2015. For example, the combination of daratumumab with bortezomib and dexamethasone improved progression-free survival in patients with RRMM compared to just bortezomib and dexamethasone [128]. Panobinostat, MS-275, and ACY1215 enhance CD38 expression, thereby increasing daratumumab's anti-myeloma effectiveness [129, 130]. In general, it has shown promising results in vitro, underscoring the potential for HDACis to improve the efficacy of established therapies in both initial and relapse settings. Despite these advancements, the specific molecular mechanisms underlying the enhanced anti-tumor activity of these combination therapies remain to be fully elucidated. Furthermore, the development of isoform and/or class-selective HDACis presents a promising avenue to mitigate the adverse effects commonly associated with non-selective HDACis, while maintaining robust anti-tumor efficacy.

The ongoing challenge of addressing toxicity, resistance mechanisms, and the absence of reliable biomarkers for predicting HDACis response underscores the need for continued research. Efforts to identify predictive markers, understand the molecular basis of HDACis action, and explore novel therapeutic combinations are essential for optimizing MM treatment. As research progresses, it is anticipated that the targeted application of HDACis, either as monotherapies or in combination with other agents, will significantly advance the treatment paradigm for MM, offering patients more personalized and effective treatment options [131, 132]. This integration of novel HDACis-based therapies into MM treatment regimens not only reflects the current progress but also sets the stage for future advancements that promise to further improve patient survival and quality of life.

## Data Availability

Not applicable.

## Abbreviations

HDACs: Histone deacetylases
HDACis: Histone deacetylase inhibitors
MM: Multiple myeloma
IMiDs: Immunomodulatory drugs
ASCT: Autologous stem cell transplantation
mAbs: Monoclonal antibodies
ADC: Antibody–drug conjugates
BiTE: Bispecific T-cell engagers
CAR-T: Chimeric antigen-T-cell therapy
FDA: The United States Food and Drug Administration
RRMM: Relapsed/refractory multiple myeloma
CTCL: Cutaneous T-cell lymphoma
CMML: Chronic myelomonocytic leukemia
HIV: Human immunodeficiency virus
AIDS: Acquired immunodeficiency syndrome
DLBCL: Diffuse large B-cell lymphoma
PTCL: Peripheral T-cell lymphoma
NSCC: Non-small cell lung carcinoma
HCC: Hepatocellular carcinoma
ATLL: Adult T-cell leukemia-lymphoma
hERG: The human ether-a-go-go
ECG: Electrocardiogram
tdP: Torsades de pointes
CIK: Cytokine-induced killer
